# Integrated GNSS Attitude Determination and Positioning for Direct Geo-Referencing

**DOI:** 10.3390/s140712715

**Published:** 2014-07-17

**Authors:** Nandakumaran Nadarajah, Jens-André Paffenholz, Peter J. G. Teunissen

**Affiliations:** 1 Global Navigation Satellite System (GNSS) Research Centre, Department of Spatial Sciences, Curtin University, GPO Box U1987, Perth WA 6845, Australia; E-Mails: N.Nadarajah@curtin.edu.au (N.N.); P.Teunissen@curtin.edu.au (P.J.G.T.); 2 Geodetic Institute, Leibniz Universität Hannover, Nienburger Str. 1, 30167 Hannover, Germany; 3 Delft Institute of Earth Observation and Space Systems (DEOS), Delft University of Technology, PO Box 5058, 2600 GB Delft, The Netherlands

**Keywords:** global navigation satellite system (GNSS), attitude determination, multivariate constrained integer least-squares (MC-LAMBDA), carrier phase ambiguity resolution, direct geo-referencing, laser scanner

## Abstract

Direct geo-referencing is an efficient methodology for the fast acquisition of 3D spatial data. It requires the fusion of spatial data acquisition sensors with navigation sensors, such as Global Navigation Satellite System (GNSS) receivers. In this contribution, we consider an integrated GNSS navigation system to provide estimates of the position and attitude (orientation) of a 3D laser scanner. The proposed multi-sensor system (MSS) consists of multiple GNSS antennas rigidly mounted on the frame of a rotating laser scanner and a reference GNSS station with known coordinates. Precise GNSS navigation requires the resolution of the carrier phase ambiguities. The proposed method uses the multivariate constrained integer least-squares (MC-LAMBDA) method for the estimation of rotating frame ambiguities and attitude angles. MC-LAMBDA makes use of the known antenna geometry to strengthen the underlying attitude model and, hence, to enhance the reliability of rotating frame ambiguity resolution and attitude determination. The reliable estimation of rotating frame ambiguities is consequently utilized to enhance the relative positioning of the rotating frame with respect to the reference station. This integrated (array-aided) method improves ambiguity resolution, as well as positioning accuracy between the rotating frame and the reference station. Numerical analyses of GNSS data from a real-data campaign confirm the improved performance of the proposed method over the existing method. In particular, the integrated method yields reliable ambiguity resolution and reduces position standard deviation by a factor of about 0.8, matching the theoretical gain of $\sqrt{3/4}$ for two antennas on the rotating frame and a single antenna at the reference station.

## 1. Introduction

The acquisition and interpretation of three-dimensional (3D) spatial data are important assets for scientific and industrial applications, such as 3D city modeling, facility management, construction engineering, navigation and forensic investigations. Direct geo-referencing, which does not require dedicated control points, is an efficient methodology for the fast acquisition of 3D spatial data by means of a 3D laser scanner. It can be performed either using additional backsight targets [1–4] or using external sensors [5–8]. The latter requires the fusion of spatial data acquisition sensors and navigation sensors, such as Global Navigation Satellite System (GNSS) sensors. In this contribution, we consider an integrated GNSS navigation system to provide estimates of the position and attitude (orientation) of a 3D laser scanner.

The use of GNSS for geo-referencing has been explored in various studies. Direct geo-referencing is demonstrated using GNSS integrated with inertial sensors [9,10] and a digital compass [5]. In this work, we explore pure a GNSS-based navigation solution as in [8,11]. In [11], a single rotating antenna is used to provide a post-processing navigation solution. As in [7,8], the proposed multi-sensor system (MSS) consists of multiple GNSS antennas rigidly and symmetrically mounted on the frame of a rotating laser scanner and a reference GNSS station with known coordinates.

The proposed method uses the multivariate constrained integer least-squares (MC-LAMBDA) method [12–17] for the estimation of rotating frame ambiguities and attitude angles. MC-LAMBDA makes use of known antenna geometry to strengthen the underlying attitude model, enabling reliable instantaneous ambiguity resolution and attitude determination of the rotating frame. The reliable estimation of rotating frame ambiguities is consequently utilized to enhance the positioning of the rotating frame. This array-aided positioning method [15,18–20] naturally yields the estimates of the rotating frame center (centroid of antennas' reference points) and improves ambiguity resolution, as well as the positioning accuracy of the relative position between the rotating frame and the reference station.

The numerical studies considered in this contribution include performance analyses of the proposed method with GNSS data from two real data campaigns. Comparison studies using epoch-by-epoch processing and filtering confirm the improved performance of the proposed method over the existing method from [8]. This contribution is organized as follows: Section 2 describes the multi-sensor system considered and defines the problem. Section 3 describes our attitude determination and filtering approaches for the rotating frame. Section 4 describes the array-aided positioning and filtering methods for the positioning of the rotating frame. Section 5 presents real data analyses demonstrating the improved performance of the proposed method. Finally, Section 6 contains the summary and conclusions of this contribution.

## 2. Background

**Figure 1.**
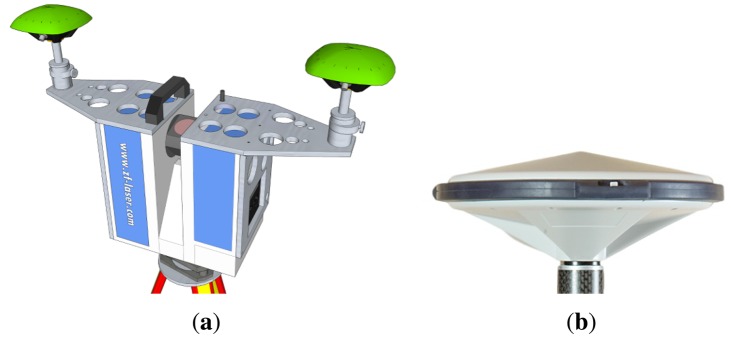
Geo-referencing scenario. (**a**) The MSS formed by a laser scanner (blue) and two eccentrically-mounted GNSS antennas (green) [8]; and (**b**) the reference GNSS station with a known position.

The multi-sensor system (MSS) considered for geo-referencing in this contribution consists of a laser scanner and two GNSS antennas/receivers. As shown in Figure 1a, the laser scanner is the core sensor of the MSS, which rotates about its vertical axis with a constant angular velocity. GNSS receivers are connected to two eccentric GNSS antennas, which are mounted such that the centroid of the antenna reference points (ARPs) coincides with the scanner rotating axis. In addition to these GNSS receivers, it is assumed to have a nearby reference GNSS station with a known position (Figure 1b). During the data acquisition, the MSS makes a complete 360 degree rotation about its vertical axis, collecting both laser and GNSS measurements, which are synchronized through a GNSS receiver event marker.

The objective of the navigation system is to provide the position (the centroid of ARPs) and the pointing direction (heading) of the laser scanner. In [8], standard real-time kinematic (RTK) positioning [21] is used to estimate individual rotating antenna positions, and then, a constrained nonlinear filtering method, in particular an extended Kalman filter, is used to obtain the above parameters. In this contribution, we use constrained integer least-squares (Section 3) and array-aided positioning (Section 4), enabling improved ambiguity resolution and improved positioning accuracy. In the following sections, we formulate a more general problem, estimating attitude angles and relative position between two platforms with multiple GNSS antennas/receivers, which enables us to demonstrate the potential of array-aided positioning. As shown in the following sections, array-aided positioning utilizes the reliable estimation of rotating frame ambiguities, which are obtained from array processing (MC-LAMBDA), improving the estimation of the relative position of the rotating frame with respect to the given reference station.

## 3. Attitude Determination

This section describes the platform processing involving attitude determination for a small-sized array of GNSS receivers/antennas with a known local body frame antenna geometry First, the multi-baseline attitude model is introduced using the multivariate formulation of [12]. This formulation makes frequent use of the Kronecker product and the vec-operator [22]. Then, we include the local body frame antenna-geometry and show how the constrained attitude model can be solved in a step-wise manner.

### 3.1. The Multivariate Model

Let us consider the *k*-th platform equipped with a set of *n_k_* + 1 antennas simultaneously tracking *m* + 1 satellites on *f* frequencies. The set of linearized double difference (DD) GNSS phase and code observations obtained on the *n_k_* baselines formed by these antennas at an observation epoch forms a multivariate Gauss–Markov model [12,19]:

(1) $${\begin{array}{ccc} \text{E}\left(Y^{k}\right) & =AZ^{k}+GB^{k}, & Z^{k}\in \text{ℤ} \end{array}}^{fm\times n_{k}}$$

(2) $$\begin{array}{cc} \text{D}\left(\text{vec}\left(Y^{k}\right)\right)=Q_{Y^{k}Y^{k}}={P_{n}}_{k}⊗Q_{yy}, & B^{k}\in {\text{ℝ}}^{3\times n_{k}} \end{array}$$

where E(·) and D(·) denote the expectation and dispersion operator, ⊗ denotes the Kronecker product, $Y^{k}=[y_{1}^{k},\ldots ,y_{n_{k}}^{k}]$ the 2*fm* × *n_k_* matrix of *n_k_* linearized (observed-minus-computed) DD observation vectors $y_{r}^{k}$, $Z^{k}=[z_{1}^{k},\ldots ,z_{n_{k}}^{k}]$ the *fm* × *n_k_* matrix of *n_k_* unknown DD integer ambiguity vectors $z_{j}^{k}$, $B^{k}=[b_{1}^{k},\ldots ,b_{n_{k}}^{k}]$ the 3 × *n_k_* matrix of *n_k_* unknown baseline vectors *b_j_*, *G* the 2*fm* × 3 geometry matrix that contains the unit line-of-sight vectors, *A* the 2*fm* × *fm* matrix that links the DD data to the integer ambiguities and *P_n_k__* and *Q_yy_* the known matrices of order *n_k_* × *n_k_* and 2*fm* × 2*fm*, respectively. Here, vec(·) denotes the vec-operator, which transforms a matrix into a vector by stacking the columns of the matrix, one underneath the other. Note that, for the simplicity of the formulation, we assumed that all receivers/antennas track the same set of satellites. However, this restriction is relaxed in the software implemented using MATLAB. Since the unit line-of-sight vectors of two antennas to the same satellite on a short baseline considered in this work (≤ 10 km) are the same for all practical purposes, the geometry matrix *G* is considered the same for different platforms, as well as for the between-platform baseline at a given time instant.

For the stochastic model, we assumed that all receivers/antennas have similar (noise) characteristics. However, the results in the following are also applicable for dissimilar receivers/antennas [19]. The correlation matrix *P_n_k__* takes care of the correlation that follows from the fact that the *n_k_* baselines share the observations from the reference receiver. For similar receivers/antennas, it is given as:

(3) $$P_{n_{k}}=\frac{1}{2}(I_{n_{k}}+e_{n_{k}}e_{n_{k}}^{T})$$

with *I_n_k__* the identity matrix of size *n_k_* and *e_n_k__* the *n_k_*-vector of ones. Matrix *Q_yy_* takes care of the precision of the phase and code data and is given as:

(4) $$Q_{yy}=\text{blockdiag}\left(Q_{1},\ldots ,Q_{f}\right)$$

where:

(5) (5) $$Q_{f}=2\times \text{blockdiag}\left(Q_{f:p},Q_{f:\Phi }\right)$$

with $Q_{f:p}=D_{m}^{T}{Q^{\prime }}_{f:p}D_{m}$, $Q_{f:\Phi }=D_{m}^{T}{Q^{\prime }}_{f:\Phi }D_{m}$, $Q_{f:t}^{\prime }=\text{diag}\left[{\left(\sigma_{f:t}^{1}\right)}^{2},\ldots ,{\left(\sigma_{f:t}^{m+1}\right)}^{2}\right]$, $D_{m}^{T}=[\begin{array}{cc} -e_{m} & I_{m} \end{array}]$ the single difference operator, “blockdiag” referring to the block diagonal matrix formed by given arguments and “diag” referring to the diagonal matrix formed by given arguments. The factor two in Equation (5) is due to the between-receiver difference of similar receivers. We assume elevation-dependent noise characteristics [23] for the undifferenced observables. That is, the standard deviation of the undifferenced observable can be written as:

(6) $$\sigma_{f:t}^{s}=\sigma_{f:t_{0}}\left(1+a_{f:t_{0}}\exp\left(\frac{-\theta^{s}}{\theta_{f:t_{0}}}\right)\right)$$

where *θ^s^* is the elevation angle of satellite *s* and *σ*_*f:t*_0__, *a_f_*_:__*t*_0__ and *θ*_*f:t*_0__ are the elevation-dependent model parameters.

### 3.2. The Body-Frame Antenna-Geometry as Multivariate Constraints

The strength of the above model can be improved by including information about the geometry of the antenna configuration. The known body-frame antenna-geometry can be included into the above model through the parametrization:

(7) $$\begin{array}{cc} B^{k}=R^{k}B_{0}^{k}, & R^{k}\in {\text{O}}^{3\times q_{k}} \end{array}$$

with the unknown 3×*q_k_* orthogonal matrix $R^{k}=(R^{k^{T}}R^{k}=I_{q_{k}})$, 
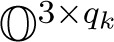
 denoting the set of orthogonal matrices of size 3 × *q_k_* and the known *q_k_* × *n_k_* matrix $B_{0}^{k}=[b_{0,1}^{k},\ldots ,b_{0,n_{k}}^{k}]$ describing the known geometry of the antenna configuration in the body frame. Here, *q_k_* is the degree of geometrical independence of the GNSS baselines, for example, *q_k_* = 1 for co-linearly installed antennas, *q_k_* = 2 for co-planarly installed antennas and *q_k_* = 3 for antennas not installed in a single plane. For *q_k_* = 3, *R^k^* is related to the Euler attitude angles *ϑ* = [*ϕ θ ψ*]*^T^* as follows:

(8) $$R(\vartheta )=\left[\begin{array}{lll} c_{\theta }c_{\phi } & -c_{\psi }s_{\phi }+s_{\psi }s_{\theta }c_{\phi } & s_{\psi }s_{\phi }+c_{\psi }s_{\theta }c_{\phi }\\ c_{\theta }s_{\phi } & c_{\psi }c_{\phi }+s_{\psi }s_{\theta }s_{\phi } & -s_{\psi }c_{\phi }+c_{\psi }s_{\theta }c_{\phi }\\ -s_{\theta } & s_{\psi }c_{\theta } & c_{\psi }c_{\theta } \end{array}\right]$$

with *ϕ* the heading, *θ* the elevation, *ψ* the bank and where *s_α_* = sin(*α*) and *c_α_* = cos(*α*). Note that for *q* < 3, only the first *q* columns of *R* are defined. For example, for a linear antenna array (*q* = 1), only the first column is defined, and hence, only heading and elevation are estimable. For *q* > 1 (an array with more than two antennas that are not in a straight line), all three angles are estimable.

Substitution of Equation (7) into Equation (1) leads to the constrained GNSS attitude model [19,24]:

(9) $$\begin{array}{cccc} \text{E}\left(Y^{k}\right) & = & AZ^{k}+GR^{k}B_{0}^{k} & Z^{k}\in {\mathbb{Z}}^{fm\times n_{k}} \end{array}$$

(10) $$\begin{array}{cccc} \text{D}\left(\text{vec}\left(Y^{k}\right)\right) & = & Q_{Y^{k}Y^{k}}=P_{n_{k}}⊗Q_{yy} & R^{k}\in {\text{O}}^{3\times q_{k}} \end{array}$$

Our objective is to solve for the attitude matrix *R^k^* in a least-squares sense, thereby taking the integer constraint on matrix *Z^k^* ∈ ℤ*^fm^*^×^*^nk^* and the orthonormality constraint on matrix *R^k^* ∈ 
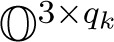
 into account. Hence, the least-squares minimization problem that will be solved reads:

(11) $$\underset{Z^{k}\in {\mathbb{Z}}^{fm\times n_{k}},R^{k}\in {\text{O}}^{3\times q_{k}}}{\min}{\left\|\text{vec}\left(Y^{k}-AZ^{k}-GR^{k}B_{0}^{k}\right)\right\|}_{Q_{Y^{k}Y^{k}}}^{2}$$

with ${\left\|\cdot \right\|}_{Q}^{2}={\left(\cdot \right)}^{T}Q^{-1}\left(\cdot \right)$. This is a mixed integer nonlinear least-squares problem that does not permit a closed-form solution. We now describe how Equation (11) can be solved.

### 3.3. The Real-Valued Float Solution

The float solution is defined as the solution of Equation (11) without the constraints. When we ignore the integer constraint on *Z^k^* and the orthonormality constraint on *R^k^*, the float solutions *Ẑ^k^* and *R̂^k^* and their variance-covariance matrices are obtained from solving the:

(12) $${\left[\begin{array}{cc} Q_{{\hat{Z}}^{k}{\hat{Z}}^{k}} & Q_{{\hat{Z}}^{k}{\hat{R}}^{k}}\\ Q_{{\hat{R}}^{k}{\hat{Z}}^{k}} & Q_{{\hat{R}}^{k}{\hat{R}}^{k}} \end{array}\right]}^{-1}\left[\begin{array}{c} \text{vec}({\hat{Z}}^{k})\\ \text{vec}({\hat{R}}^{k}) \end{array}\right]=A_{k}^{T}Q_{Y^{k}Y^{k}}^{-1}\text{vec}(Y^{k})$$

with:

$$\left[\begin{array}{cc} Q_{{\hat{Z}}^{k}{\hat{Z}}^{k}} & Q_{{\hat{Z}}^{k}{\hat{R}}^{k}}\\ Q_{{\hat{R}}^{k}{\hat{Z}}^{k}} & Q_{{\hat{R}}^{k}{\hat{R}}^{k}} \end{array}\right]={\left(A_{k}^{T}Q_{Y^{k}Y^{k}}^{-1}A_{k}\right)}^{-1},A_{k}={\left[\begin{array}{c} I_{n_{k}}⊗A^{T}\\ B_{0}^{k}⊗G^{T} \end{array}\right]}^{T}$$

The *Z^k^* -c of *R^k^* and its variance-covariance matrix can be obtained from the float solution as follows:

(13) $$\text{vec}\left({\hat{R}}^{k}\left(Z^{k}\right)\right)=\text{vec}({\hat{R}}^{k})-Q_{{\hat{R}}^{k}{\hat{Z}}^{k}}Q_{{\hat{Z}}^{k}{\hat{Z}}^{k}}^{-1}\text{vec}\left({\hat{Z}}^{k}-Z^{k}\right)$$

(15) $$\begin{array}{ll} Q_{{\hat{R}}^{k}(Z^{k}){\hat{R}}^{k}(Z^{k})} & =Q_{{\hat{R}}^{k}{\hat{R}}^{k}}-Q_{{\hat{R}}^{k}{\hat{Z}}^{k}}Q_{{\hat{Z}}^{k}{\hat{Z}}^{k}}^{-1}Q_{{\hat{Z}}^{k}{\hat{R}}^{k}}\\ & ={\left(B_{0}^{k}{P_{n_{k}}}^{-1}B_{0}^{k^{T}}\right)}^{-1}⊗{\left(G^{T}Q_{yy}^{-1}G\right)}^{-1} \end{array}$$

Using the above estimators, the original problem in Equation (11) can be decomposed as:

(15) $$\begin{array}{c} \underset{Z^{\text{k}}\in {\mathbb{Z}}^{fm\times n_{k}},{\text{R}}^{k}\in {\text{O}}^{3\times q_{k}}}{\min}{\left\|\text{vec}\left(Y^{k}-AZ^{k}-GR^{k}B_{0}^{k}\right)\right\|}_{Q_{Y^{k}Y^{k}}}^{2}\\ ={\left\|\text{vec}\left({\hat{E}}^{k}\right)\right\|}_{Q_{Y^{k}Y^{k}}}^{2}+\underset{Z^{k}\in {\mathbb{Z}}^{fm\times n_{k}}}{\min}({\left\|\text{vec}\left({\hat{Z}}^{k}-Z^{k}\right)\right\|}_{Q_{{\hat{Z}}^{k}{\hat{Z}}^{k}}}^{2}\\ +\underset{R^{k}\in {\text{O}}^{3\times q_{k}}}{\min}{\left\|\text{vec}\left({\hat{R}}^{k}(Z^{k})-R^{k}\right)\right\|}_{Q_{\hat{R}}k(Z^{k}){\hat{R}}^{k}(Z^{k})}^{2}) \end{array}$$

with ${\hat{E}}^{k}=Y^{k}-A{\hat{Z}}^{k}-G{\hat{R}}^{k}B_{0}^{k}$ being the matrix of least-squares residuals. Note that the first term on the right-hand side is constant, as it does not depend on the unknown matrices *Z^k^* and *R^k^*.

### 3.4. The Integer Ambiguity Solution

Based on the orthogonal decomposition (15), the multivariate constrained integer minimization can be formulated as:

(16) $${\overset{⌣}{Z}}^{k}=\arg\underset{Z^{k}\in {\mathbb{Z}}^{fm\times n_{k}}}{\min}C^{k}(Z^{k})$$

where:

(17) $$C^{k}\left(Z^{k}\right)={\left\|\text{vec}\left({\hat{Z}}^{k}-Z^{k}\right)\right\|}_{Q_{{\hat{Z}}^{k}{\hat{Z}}^{k}}}^{2}+{\left\|\text{vec}\left({\hat{R}}^{k}\left(Z^{k}\right)-{\overset{⌣}{R}}^{k}\left(Z^{k}\right)\right)\right\|}_{Q_{{\hat{R}}^{k}(Z^{k}){\hat{R}}^{k}(Z^{k})}}^{2}$$

with:

(18) $${\overset{⌣}{R}}^{k}(Z^{k})=\arg\underset{R^{k}\in {\text{O}}^{3\times q_{k}}}{\min}{\left\|\text{vec}\left({\hat{R}}^{k}(Z^{k})-R^{k}\right)\right\|}_{Q_{{\hat{R}}^{k}(Z^{k}){\hat{R}}^{k}(Z^{k})}}^{2}$$

The ambiguity objective function *C^k^*(*Z^k^*) is the sum of two coupled terms: the first weighs the distance from the float ambiguity matrix *Ẑ^k^* to the nearest integer matrix *Z^k^* in the metric of *Q_Ẑ^k^Ẑ^k^_*, while the second weighs the distance from the conditional float solution *R̂^k^*(*Z^k^*) to the nearest orthonormal matrix *R^k^* in the metric of *Q_R̂^k^_*_(_*_Z^k^_*_)_ *_R̂^k^_*_(_*_Z^k^_*_)_. Unlike with the standard LAMBDA method [25], the search space of the above integer minimization problem is non-ellipsoidal, due to the presence of the second term in *C^k^*(*Z^k^*). This second term is a consequence of having the orthonormality constraints rigorously included. The evaluation of *C^k^*(*Z^k^*) requires the computation of a nonlinear constrained least-squares problem (18) for every integer matrix in the search space. In the MC-LAMBDA method, this problem is mitigated through the use of easy-to-evaluate bounding functions [17].

### 3.5. The Ambiguity Resolved Attitude Solution

Finally, we obtain the integer ambiguity-resolved attitude solution by substituting Z*˘^k^* into Equation (13), thus giving *R̂^k^*(*Z̆^k^*). The sought-for attitude angles ϑ*^k^* (*Z̆^k^*) are then given by the reparametrized solution of Equation (18). Using a first order approximation, the formal variance-covariance matrix of the attitude angles is given by:

(19) $$Q_{\vartheta^{k}\vartheta^{k}}\approx {\left(J_{R^{k},\vartheta^{k}}^{T}Q_{{\hat{R}}^{k}(Z^{k}){\hat{R}}^{k}(Z^{k})}^{-1}J_{R^{k},\vartheta^{k}}\right)}^{-1}$$

where *J_R^k^_*_,_*_ϑ^k^_* is the Jacobian of ϑ*^k^*(*R^k^*).

### 3.6. Attitude Filtering

The epoch-by-epoch MC-LAMBDA attitude solution is further processed using an unscented Kalman filter (UKF) [26]. For a leveled platform (*i.e.*, for small *θ* and *ψ*), the kinematic equations of the attitude angles are given as [27]:

(20) $$\alpha_{i}=F\alpha_{i-1}+\upsilon_{i-1}^{\alpha }$$

where the state vector *α_i_* = [*ϕ_i_ ϕ ˙_i_ θ_i_* *θ̇_i_ ψ_i_ ψ̇_i_*]*^T^* consists of attitude angles and angular rates, and the state transition matrix *F* is given as:

(21) $$F=I_{3}⊗\left[\begin{array}{cc} 1 & T\\ 0 & 1 \end{array}\right]$$

where *T* is the sampling interval. The process noisev $\upsilon_{i-1}^{\alpha }$ has a zero mean normal distribution with variance-covariance matrix *Q_υ^α^υ^α^,i_*_−1_ which is given as:

(22) $$Q_{\upsilon^{\alpha }\upsilon^{\alpha },i-1}=\text{diag}\left(\left[\sigma_{\phi }^{2},\sigma_{\theta }^{2},\sigma_{\psi }^{2}\right]\right)⊗\left[\begin{array}{cc} T^{3}/3 & T^{2}/2\\ T^{2}/2 & T \end{array}\right]$$

with *σ_ϕ_*, *σ_θ_* and *σ_ψ_* the process noise standard deviations. The observation model reads:

(23) $$\zeta_{i}=h(\alpha_{i})+w_{i}^{\alpha }$$

with *ζ_i_* given by *R̂^k^*(*Z̆^k^*)) at epoch *i*. The nonlinear observational function *h*(*α_i_*) is defined by Equation (8), and the observation noise $w_{i}^{\alpha }$ is assumed to have a zero mean normal distribution with covariance matrix *Q_w^α^w^α^,i_*, Q*_R̂^k^(Z̆^k^)R̂^k^(Z̆^k^)_* at epoch *i*.

**Figure 2.**
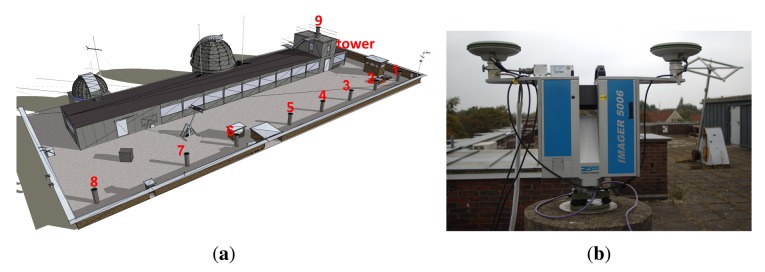
Multi-sensor experiment set-up on the roof of the building of the Geodetic Institute (Messdach), Leibniz Universität Hannover, Germany. The MSS is mounted on Pillar 5, while the reference station is located on Pillar 8. (**a**) Location; and (**b**) the MSS used in the experiments.

The use of the above constant-velocity model [28] reflects the fact that the frame is rotating at a constant rate. For the two-antenna scenario considered in real-data analyses (Figure 2), only heading and elevation angles are estimable. Hence, a reduced state space model consisting of only heading, elevation and their rates is used in Section 5. The recursive filter is initialized with two-point initialization [28] and propagated with process noise standard deviations of $\sigma_{\phi }=0.01{}^{\circ}{\text{s}}^{-\frac{3}{2}}$ and $\sigma_{\theta }=0{}^{\circ}{\text{s}}^{-\frac{3}{2}}$ (*i.e.*, dead reckoning filtering for elevation constraining to horizontal 1D rotation).

## 4. Integrated Positioning

This section describes the between-platform processing involving relative positioning between two platforms equipped with arrays of GNSS receivers/antennas. The array-aided positioning described in the following is a novel positioning concept improving between-platform positioning using an array of antennas on the platforms [15,18–20]. Unlike the formulations in [18–20], the formulation in this contribution explicitly considers different numbers of antennas on the reference and user platforms. Unlike the parameter space formulation in [15], the current contribution considers a simplified, double-difference observation space formulation elegantly demonstrating the advantages of array-aided positioning. First, the combined observation model for all independent baselines among all receivers on both platforms is described. Then, we describe attitude-bootstrapping, showing how platform arrays improve the between-platform baseline estimate.

Let us consider two platforms carrying *n*_1_ + 1 and *n*_2_ + 1 receivers/antennas. The functional and stochastic models for the between-platform baseline formed by the first antennas (pivot antennas) read:

(24) $$\begin{array}{ccc} \text{E}\left(y^{12}\right) & =Az^{12}+Gb^{12} & z^{12}\in {\mathbb{Z}}^{fm} \end{array}$$

(25) $$\begin{array}{cc} \text{D}(y^{12}) & =Q_{yy} \end{array}$$

where *y*^12^ is the between-platform double-difference observables, *z*^12^ is the unknown between-platform double-difference ambiguities and *b*^12^ is the unknown between-platform baseline. Note that atmosphere delays are not considered in this formulation, as troposphere delays and ionosphere delays can be ignored for the short baseline (<10 km) considered in this work. However, these atmosphere delays must be taken into account for general long baseline scenarios [19]. In standard positioning, the LAMBDA method yields the optimal estimates for the ambiguities and, hence, for the baseline.

### 4.1. Integrated Between-Platform Model

By combining between-platform observables in Equation (24) and platform array observables in Equation (9), the functional and stochastic models of the integrated system read:

(26) $$\begin{array}{cc} E(Y) & =AZ+GR \end{array}B_{0}$$

(27) $$\begin{array}{cc} \text{D}\left(\text{vec}(Y)\right) & =P⊗Q_{yy} \end{array}$$

where 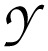 = [*Y*^1^ *Y*^2^ *y*^12^] is the combined observation matrix, 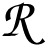 = [*R*^1^ *R*^2^ *b*^12^] ∈ ℝ^3×(^*^q^*^1+q2+1)^ is the combined rotation matrices and between-platform baseline, 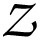 = [*Z*^1^ *Z*^2^ *z*^12^] ∈ ℤ*^fm^*^×^*^nt^* is the combined ambiguity matrix, *B*_0_ = blockdiag ( $B_{0}^{1}$, $B_{0}^{2}$, 1) is the combined local geometry matrix and:

(28) $$P=\left[\begin{array}{ccc} P_{n_{1}} & 0 & \frac{1}{2}e_{n_{1}}\\ 0 & P_{n_{2}} & -\frac{1}{2}e_{n_{2}}\\ \frac{1}{2}e_{n_{1}}^{T} & -\frac{1}{2}e_{n_{2}}^{T} & 1 \end{array}\right]$$

is the combined correlation matrix with *n_t_* = *n*_1_+*n*_2_ +1. The above system consists of attitude models of both platforms with unknowns *Z^k^* and *R^k^* and the between-platform baseline model with unknowns *z^12^* and *b*^12^. Even though these three subsystems do not have any parameter in common, they are correlated as in Equation (28), due to the use of common observations from pivot antennas.

### 4.2. Attitude Bootstrapping

The attitude bootstrapping method [18,19] uses a decorrelation technique to decouple the combined system in Equation (26), such that the subsystems still yield the optimal solution. Using decorrelation matrix:

(29) $$D=\left[\begin{array}{ccc} I_{n_{1}} & 0 & 0\\ 0 & I_{n_{2}} & 0\\ -\frac{1}{2}e_{n_{1}}^{T}P_{n_{1}}^{-1} & \frac{1}{2}e_{n_{2}}^{T}P_{n_{2}}^{-1} & 1 \end{array}\right]⊗I_{2fm}$$

The decorrelated system reads:

(30) $$E(Y^{\prime })=AZ^{\prime }+GR^{\prime }B_{0}$$

(31) $$\text{D}\left(\text{vec}(Y^{\prime })\right)=P^{\prime }⊗{\text{Q}}_{yy}$$

where 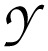
*′* = [*Y*^1^ *Y*^2^ *y′*^12^] is the decorrelated observation matrix, 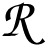
*′* = [*R*^1^ *R*^2^ *b′*^12^] ∈ ℝ^3×(^*^q^*^1+^*^q^*^2+1)^ is the combined rotation matrices and between-platform baseline after decorrelation, 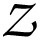
*′* = [*Z*^1^ *Z*^2^ *z′*^12^] is the combined ambiguity matrix after decorrelation and:

(32) $$P^{\prime }=\text{blockdiag}(P_{n1},P_{n2},\eta )$$

with:

(34) $${y^{\prime }}^{12}=y^{12}-\frac{1}{n_{1}+1}\sum_{r=1}^{n_{1}}y_{r}^{1}+\frac{1}{n_{2}+1}\sum_{r=1}^{n_{2}}y_{r}^{2}$$

(34) $${z^{\prime }}^{12}=z^{12}-\frac{1}{n_{1}+1}\sum_{r=1}^{n_{1}}z_{r}^{1}+\frac{1}{n_{2}+1}\sum_{r=1}^{n_{2}}z_{r}^{2}$$

(35) $${b^{\prime }}^{12}=b^{12}-\frac{1}{n_{1}+1}\sum_{r=1}^{n_{1}}R^{1}b_{0,r}^{1}+\frac{1}{n_{2}+1}\sum_{r=1}^{n_{2}}R^{2}b_{0,r}^{1}$$

(36) $$\eta =\frac{n_{t}+1}{2(n_{1}+1)(n_{2}+1)}$$

This decorrelation keeps the platform processing intact as in Equation (16) and only alters the between-platform model. As a result of decorrelation, the ambiguities in Equation (34) may not be an integer. However, once platform ambiguities are determined reliably using MC-LAMBDA with decoupled platform models in Equation (30), the model for the between-platform baseline can be rearranged as:

(37) $$\begin{array}{ccc} \text{E}({y^{\prime \prime }}^{12}) & =Az^{12}+G{b^{\prime }}^{12} & z^{12}\in {\mathbb{Z}}^{fm} \end{array}$$

(38) $$\begin{array}{ccc} \text{D}\left({y^{\prime \prime }}^{12}\right) & = & \eta Q_{yy} \end{array}$$

where:

(39) $${y^{\prime \prime }}^{12}={y^{\prime }}^{12}+\frac{1}{n_{1}+1}\sum_{r=1}^{n_{1}}Az_{r}^{1}-\frac{1}{n_{2}+1}\sum_{r=1}^{n_{2}}Az_{r}^{2}$$

Due to the reduction of variance-covariance by a factor of *η*, this model yields improved ambiguity resolution and baseline estimation compared to the standard positioning model in Equation (24). That is, the use of array-aided positioning reduces the variance-covariance matrices of the float ambiguities and ambiguity-fixed baseline estimators by a factor of *η*. For the rotating frame with two antennas and a single antenna at the reference station in Figure 1, the variance reduction factor is $\eta =\frac{3}{4}$. Note that the between-platform baseline estimate in Equation (35) corresponds to between-centroids of antenna arrays, naturally yielding the parameter of interest for the geo-referencing system in Figure 1. The unconstrained mixed-integer least-squares problem defined in Equations (37) and (38) can be solved efficiently using the LAMBDA method [25] providing ambiguity-fixed baseline estimate 
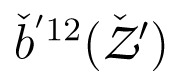
 and associated variance-covariance matrix 
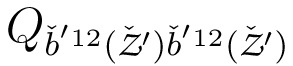
.

### 4.3. Baseline Filtering

The epoch-by-epoch baseline solution in Section 4.2 is further processed to obtain the filtered estimates for the center of the MSS (Figure 1a). Unlike the previous method in [8], which uses constrained nonlinear filtering for antenna positions, the integrated method in Section 4.2 yields estimates of the center position, which is assumed to be stationary for a rotating, leveled frame. Hence, dead reckoning (linear) Kalman filtering yields the filtered estimates for the stationary center position. The kinematic equation reads:

(40) $$\beta_{i}=\beta_{i-1}+\upsilon_{i-1}^{\beta }$$

where the state vector $\beta_{i}=b_{i}^{\prime 12}$ consists of position components. The process noise $\upsilon_{i-1}^{\beta }$ has a zero mean normal distribution with variance-covariance matrix *Q*_*υ^β^υ^β^,i*−1_, which is given as:

(41) $$Q_{\upsilon^{\beta }\upsilon^{\beta },i-1}=\text{diag}\left(\left[\sigma_{b_{x}}^{2},\sigma_{b_{y}}^{2},\sigma_{b_{z}}^{2}\right]\right)$$

with *σ_b_x__*, *σ_b_y__* and *σ_b_z__* the process noise standard deviations. Since the center position of the rotating frame is stationary, a dead reckoning filter is used (*i.e.*, *σ_b_x__* = *σ_b_y__* = *σ_b_z__* = 0, which is equivalent to the recursive least-squares estimation of constant parameter vector *b*′^12^). The observation model reads:

(42) $$\xi_{i}=\beta_{i}+w_{i}^{\beta }$$

with *ξ_i_* given by 
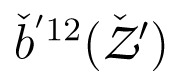
 at epoch *i*. Observation noise $w_{i}^{\beta }$ is assumed to have a zero mean normal distribution with covariance matrix *Q_w^β^w^β^,i_*, which is given by 
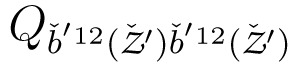
 at epoch *i*.

## 5. Analyses

For numerical analyses, we used the data from a static and a kinematic experiment on the roof of the Geodetic Institute (Messdach) building, Leibniz Universität Hannover, Germany. The MSS is mounted on Pillar 5 (*cf.* Figure 2) and equipped with a terrestrial laser scanner (TLS) Z+F Imager 5006 and two LEIAX1202GG GNSS antennas about 0.6 m apart. These antennas are connected to two JAVAD TRE_G3TH DELTA GNSS receivers. The reference station is located on Pillar 8 (about 20 m from the MSS and equipped with a JAVAD TRE_G3TH DELTA GNSS receiver and a LEIAR25.R3 LEIT antenna. For the kinematic experiment, we also considered another reference station equipped with a LEICA GRX1200 GNSS receiver and a LEIAR25.R4 LEIT antenna and located about 6 km away from the MSS.

The static experiment was conducted on 4 October 2011, for about six hours with the collection of GPS data at a rate of 1 Hz. In the kinematic experiment on 7 October 2011, we collected GPS data for five subsequent rotations (about an hour) at a rate of 10 Hz. For all of our analyses, we used the elevation-dependent stochastic model with the parameters given in Table 1.

### 5.1. Static Data

**Figure 3.**
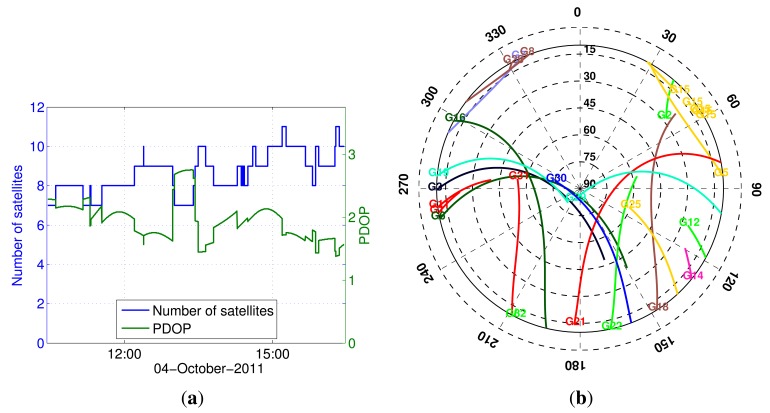
GPS satellite visibility during the static experiment with a 10° elevation cut-off. (**a**) The number of satellites and position dilution of precision (PDOP); and (**b**) skyplot.

This section presents the analyses of the static data demonstrating the benefits of using the knowledge of antenna geometry for attitude determination and array-aided positioning. Figure 3 shows the satellite visibility: the skyplot, the number of satellites and the position dilution of precision (PDOP) values. With a 10° elevation cut-off, we have a moderate GPS satellite geometry (PDOP < 3) during the experiment.

Table 2 summarizes the empirical instantaneous integer ambiguity resolution success rate for attitude determination, indicating the advantage of using MC-LAMBDA for the case of single-frequency processing. Similarly, Table 3 demonstrates the improved success rate performance of the proposed array-aided positioning with two antennas/receivers on the frame. Note that further improvement is possible by having more antennas/receivers.

**Figure 4.**
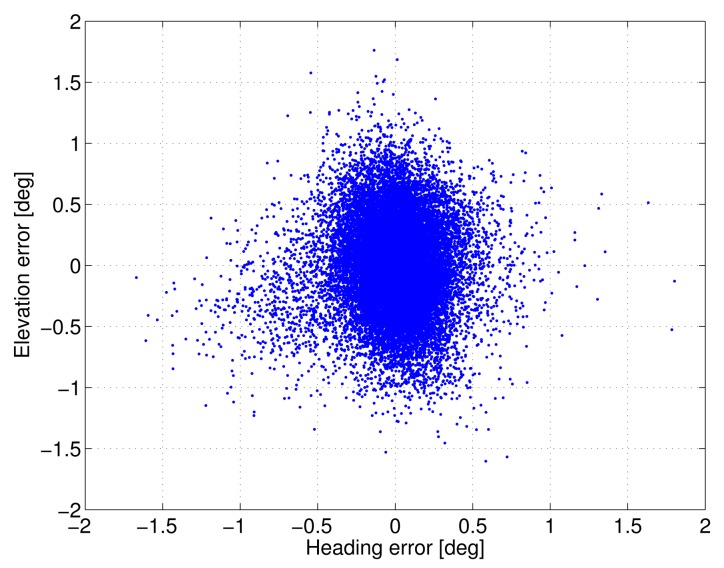
Scatter plot of the ambiguity-fixed attitude angles using epoch-by-epoch processing of the static data (0.6-m baseline).

**Figure 5.**
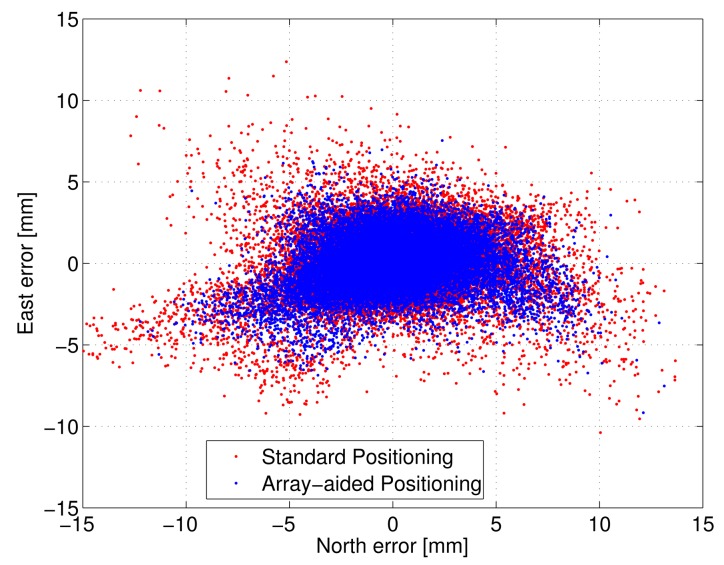
North-east scatter plot of the ambiguity-fixed baseline estimation using static data with epoch-by-epoch processing (20-m baseline).

Figure 4 depicts the scatter plot of the ambiguity-fixed attitude angles, while Figure 5 shows the plots (scatter plot of the horizontal components and time series of the down component) for the ambiguity-fixed baseline estimates. Tables 4 and 5 summarise the corresponding empirical standard deviations. Note that, once the ambiguities have been resolved, the precision of the attitude solution is driven by the high precision of the carrier phase observations [16]. That is, the accuracy of the unconstrained attitude solution (using the LAMBDA method) is comparable to that of the constrained solution (using the MC-LAMBDA method), provided that ambiguities are correctly fixed. However, the knowledge of the antenna geometry plays an important role by strengthening the underlying model and, hence, improving the ambiguity resolution (see Table 2). In the case of baseline estimation (Table 5), the proposed method yields slightly improved estimates, due to the integrated processing with an array of antennas.

### 5.2. Kinematic Data

**Figure 6.**
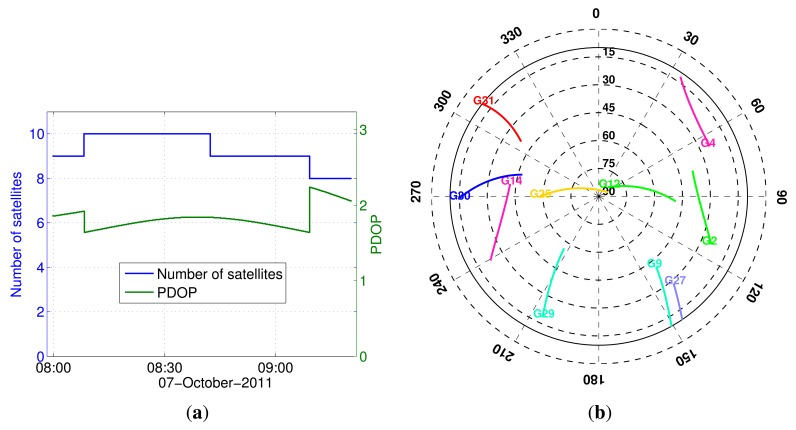
GPS satellite visibility during the kinematic experiment with a 10° elevation cut-off. (**a**) The number of satellites and PDOP; and (**b**) skyplot.

This section presents the analyses of the kinematic data comparing the proposed method with the existing method. These dual-frequency GPS data analyses aim to compare the estimation of the parameters of interest for geo-referencing, namely the heading and center point of the rotating frame. Figure 6 shows the satellite visibility: the skyplot, the number of satellites and the PDOP values. With a 10° elevation cut-off, we have a good GPS satellite geometry (PDOP ≈ 2) during the experiment.

Table 6 summarizes the root mean square (RMSE) values of the heading angle for all five rotations, where the ground truth is determined using the fact that the frame was rotating at a constant rate and synchronized though a GNSS receiver event marker. Since the precision of ambiguity-fixed attitude angles is driven by the high precision of the carrier phase observations, both the proposed method and the previous method [8] have a similar angular accuracy. Filtering further improves the estimates. Based on these analyses, the achievable heading angular accuracy using a 0.6-m baseline on the rotating frame is about 0.2° RMSE.

**Figure 7.**
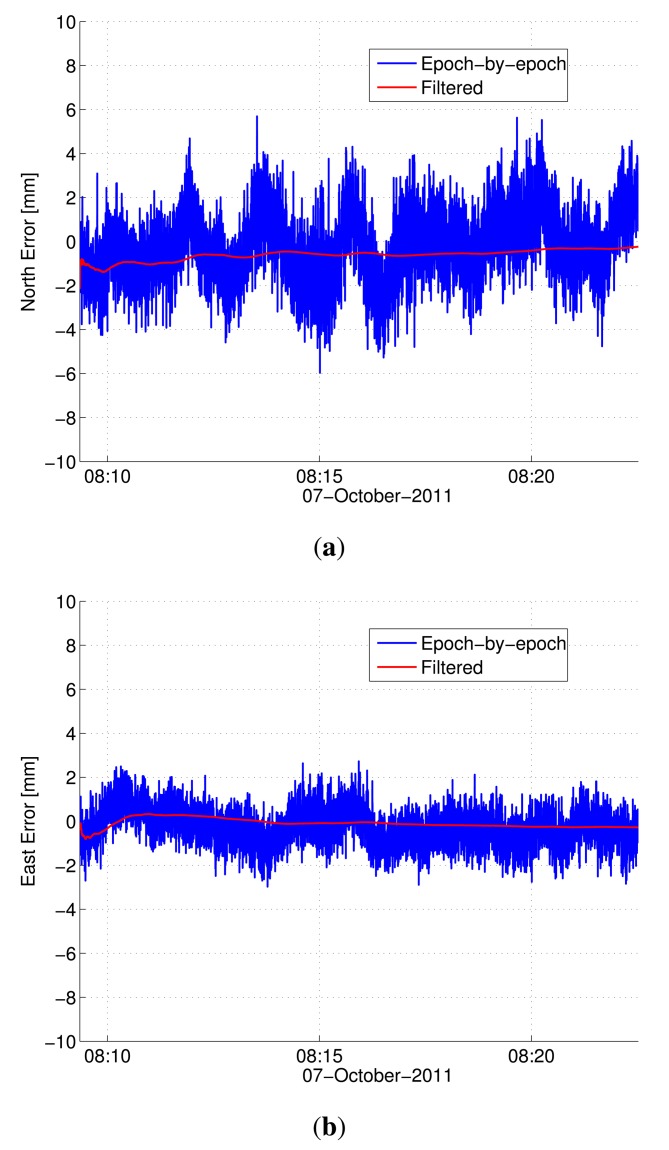
Estimation error (mm) for kinematic data (20-m baseline) using the proposed method: Rotation 1. (**a**) North error; and (**b**) east error.

Baseline estimation errors of epoch-by-epoch processing and filtering for the first rotation are depicted in Figure 7, while the 3D position RMSE values for all five rotations are reported in Table 7. Here, smoothing estimates based on all five rotations are considered as the ground truth. The apparent improved performance of the proposed method is due to the use of novel integrated processing. Since the proposed integrated method naturally yields the estimates of the center point (see Section 4.2), the proposed filtering has a simple and strong dynamic model compared to the nonlinear, constrained filtering of the existing method [8] and, hence, yields improved estimates. Based on these analyses, the achievable position accuracy using a 20-m baseline with two antennas on the rotating frame is about 3 mm RMSE.

Finally, we considered the determination of the center point using a reference station at about 6 km away. The baseline estimation results for this long baseline using the proposed method are provided in Table 8. The significant increase of the position RMSE of this baseline compared to the short baseline case in Table 7 is due to the presence of residual atmosphere delays for this long baseline. Hence, the achievable position RMSE for this baseline is about 20 mm.

## 6. Summary and Conclusions

In this contribution, we explored the use of an array of GNSS antennas for attitude determination and relative positioning for direct geo-referencing. The MC-LAMBDA method exploits the known antenna geometry to improve the reliability of resolving rotating frame ambiguities and, hence, to improve the reliability of the rotating frame attitude determination. Furthermore, the reliable estimation of rotating frame ambiguities enables the strengthening of the estimation of the baseline between the rotating frame and a reference station. Our analysis includes epoch-by-epoch processing, as well as recursive filtering. We demonstrated the improved performance of the proposed method using data from two experiments with a prototype MSS representing a rotating frame. The use of constrained attitude determination and array-aided positioning increases the reliability (in terms of ambiguity resolution) and improves the achievable position accuracy. It enables instantaneous ambiguity resolution, which is immune to cycle slips, and, hence, enables instantaneous mapping. Furthermore, the reliability and accuracy can further be improved by employing more antennas on the rotating frame and at the reference station. With a sufficient number of low-cost GNSS receivers, the potential of instantaneous mobile mapping for low-cost applications will be explored in future studies.

## Figures and Tables

**Table 1.** Elevation-dependent stochastic model parameters defined in Equation (6).

**Frequency** (*f*)	**Code**			**Phase**		

	***σ****_f_***_:_***_p_***_0_ (cm)**	***a****_f_***_:_***_p_***_0_**	***θ****_f_***_:_***_p_***_0_ (deg)**	***σ****_f_***_:_***_ϕ_***_0_** (**mm**)	***a*** *_f_***_:_***_ϕ_***_0_**	∈*_f_*_:_*_ϕ_*_0_ (**deg**)
L1 and L2	15	5	20	1	5	20

**Table 2.** Empirical instantaneous ambiguity resolution success rate (%) for attitude determination using static data (0.6-m baseline).

**Processing**	**LAMBDA**	**MC-LAMBDA**
Single-frequency	90.4	100
Dual-frequency	100	100

**Table 3.** Empirical instantaneous ambiguity resolution success rate (%) for baseline estimation using static data (20-m baseline).

**Processing**	**Standard Positioning**	**Array-Aided Positioning**
Single-frequency	85.8	90.0
Dual-frequency	100	100

**Table 4.** Empirical angular standard deviation (deg) for the attitude determination using the epoch-by-epoch processing of the static data (0.6-m baseline).

**Heading**	**Elevation**
0.24	0.38

**Table 5.** Empirical position standard deviation (mm) for baseline estimation using the epoch-by-epoch processing of the static data (20-m baseline).

**Processing Method**	**North**	**East**	**Up**
Standard positioning	3.4	2.1	5.1
Array-aided positioning	2.8	1.6	4.4

**Table 6.** Heading root mean square (RMSE) (deg) for kinematic data (0.6-m baseline).

**Rotation**	**Epoch-by-Epoch**		**Filtering**	

	**Previous Method**	**Proposed Method**	**Previous Method**	**Proposed Method**
1	0.24	0.20	0.20	0.16
2	0.26	0.23	0.24	0.20
3	0.23	0.16	0.21	0.13
4	0.20	0.15	0.16	0.11
5	0.20	0.17	0.16	0.14

**Table 7.** 3D position root mean square (RMSE) (mm) for kinematic data (20-m baseline).

**Rotation**	**Epoch-by-Epoch**		**Filtering**	

	**Previous Method**	**Proposed Method**	**Previous Method**	**Proposed Method**
1	6.1	3.1	3.5	1.5
2	4.2	2.9	3.4	1.6
3	5.5	3.2	2.9	1.5
4	5.1	3.4	3.2	1.5
5	5.3	4.7	3.4	2.6

**Table 8.** 3D position RMSE (mm) for kinematic data (6-km baseline) processing with the proposed method.

**Rotation**	**Epoch-by-Epoch**	**Filtering**
1	12.4	9.7
2	12.3	10.4
3	15.3	14.3
4	21.0	20.0
5	13.1	7.8
